# Supplementary material for: A phase division-based multi-segment foot model for estimating dynamic foot arch stiffness during walking
Source: PLoS One. 2025 Apr 23;20(4):e0320156. doi: 10.1371/journal.pone.0320156 (PMC12017521; doi:10.1371/journal.pone.0320156)
Supplement: Attachment — Submitted filename: Response to Reviewers.pdf [file pone.0320156.s001.pdf]

Responses to the Reviewers

The authors would like to thank all the editors and anonymous reviewers for their constructive comments and suggestions to help improve the quality of the paper. In the following, we list the reviewers' comments and suggestions and our responses to these feedbacks item by item.

Response to the Academic Editor

Comments to the Author:

Thank you for submitting your manuscript to PLOS ONE. After careful consideration, we feel that it has merit but does not fully meet PLOS ONE’s publication criteria as it currently stands. Therefore, we invite you to submit a revised version of the manuscript that addresses the points raised during the review process.

We appreciate the Academic Editor’s help for inviting experts to review the paper. The reviewers have offered invaluable feedback, providing a wealth of insightful queries and suggestions. In response to reviewer feedback, we have enhanced our manuscript by addressing the following: we conducted a more thorough literature review, added the rationale behind the modeling approach, clarified the result data, defined the method for calculating foot arch height, included a model accuracy validation section, addressed the limitations of the current study, and provided an outlook for future research. The technical merit of the research has been clarified and enriched as a result of this review process, consequently enhancing the manuscript's readability.

Response to Reviewer # 1

In this study, the authors presented a phase division-based multi-segment foot model that considered plantar aponeurosis's tension force for calculating the dynamics of the medial longitudinal arch. The results showed that the foot arch experienced a stiff compliant-stiff-compliant transition during a single stance phase, including an extremely low stiffness during the plantar contact phase. The method is interesting and the results are reliable, however there are few questions need to be clarified before publication.

Thanks for your careful review and suggestions for improving the manuscript. Revisions have been made according to your suggestions.

1. in the foot model part, the authors claimed that "The whole foot is divided into forefoot and rearfoot, with navicular bone as the boundary.". We usually divide foot into three parts: forefoot, midfoot and rearfoot. Why did follow the anatomy of foot? Can you remodel the foot and compare the discrepancy?

Response 1*: We appreciate your careful review and professional comments. We deeply agree that the division of the foot into forefoot, midfoot, and rearfoot is the more commonly used anatomical model in both clinical and anatomical contexts. However, forefoot-rearfoot model with the navicular as a boundary are also used in many studies.*

The reason for establishing the arch model by dividing it into forefoot and rearfoot mainly has two aspects. First, in this paper, the purpose of proposing this model is to facilitate the calculation of the loads acting on the foot arch. By dividing the foot into forefoot and rearfoot, with the arch as the dividing line, it is more effective in achieving our goal. Second, we refer to the multi-segment foot model proposed by D. A. Bruening et al. 2012 [1] (doi: 10.1016/j.gaitpost.2011.10.363), which states: "The model consists of a Shank (tibia and fibula) and three foot segments: (1) Hindfoot (calcaneus and talus), (2) Forefoot (navicular, cuboid, cuneiforms, and metatarsals), and (3) Hallux (proximal and distal phalanges). While additional segments may be useful in future work (e.g. medial/lateral and/or midfoot/forefoot segmentation), they currently present hurdles in force measurement and repeatability". We have already added this citation in the ***Foot Model*** *section: "The whole foot is divided into forefoot and rearfoot, with navicular bone as the boundary [23]."*

[1]. Bruening DA, Cooney KM, Buczek FL. Analysis of a kinetic multi-segment foot model. Part I: Model repeatability and kinematic validity. Gait & Posture. 2012;35:529 – 534.

At the same time, the models used in the numerous studies we cited that attempt to estimate the dynamic stiffness of the foot arch use the forefoot and rearfoot segmentation method. Here we list some literature in our citations:

[2]. Sanchis-Sales E, Sancho-Bru JL, Roda-Sales A, Pascual-Huerta J. Variability of the Dynamic Stiffness of Foot Joints: Effect of Gait Speed. Journal of the American Podiatric Medical Association. 2019;109:291–298.

[3]. Kern AM, Papachatzis N, Patterson JM, Bruening DA, Takahashi KZ. Ankle and midtarsal joint quasi-stiffness during walking with added mass. PeerJ. 2019;7:e7487.

[4]. Davis DJ, Challis JH. Foot arch rigidity in walking: In vivo evidence for the contribution of metatarsophalangeal joint dorsiflexion. PLoS ONE. 2022;17:e0274141.

[5]. Bruening DA, Cooney KM, Buczek FL. Analysis of a kinetic multi-segment foot model part II: Kinetics and clinical implications. Gait & Posture. 2012;35:535–540.

Certainly, many studies have incorporated the midfoot into multi-segment foot models to calculate the foot stiffness [6, 7], underscoring its important role. However, we respectfully acknowledge that it would be beyond the scope and focus of our current study to re-model the foot using the three-segment approach and compare the differences between the two methods. We have, however, included a discussion on the potential for future work using the three-segment anatomical model in the last paragraph of the **Discussion** *section: "In terms of segmentation methods for multi-segment foot models, future work includes considering the addition of the midfoot to better align the model with the anatomy of the foot, thereby improving accuracy."*

[6]. Farris DJ, Kelly LA, Cresswell AG, Lichtwark GA. The functional importance of human foot muscles for bipedal locomotion. Proceedings of the National Academy of Sciences. 2019;116:1645–1650.

[7] Zhu, S., Jenkyn, T. Development of a clinically useful multi-segment kinetic foot model. J Foot Ankle Res 16, 86 (2023). https://doi.org/10.1186/s13047-023-00686-0

2. in the table 1, did you normalized the change of arch height with arch length? Why not?

Response 2*: Thank you for your comments. There must be some misunderstanding. We are confident that both the Static Arch Height and Maximum Arch Displacement in Table 1 have been normalized*

using the Truncated Foot Length, as indicated by the units labeled "NNHt" in the table. This aligns with the explanation provided in Section ***Kinematic and Kinetic Data of Foot****, which states: "For plotting and comparison, the truncated foot length (the distance from the posterior aspect of the heel to the first metatarsal head) was used for normalization to obtain the normalized navicular height truncation (NNHt) as defined by Murley et al. [31]"*

To improve readability and assist with understanding, we have slightly revised the introductory statement in the second paragraph of the Results section, which corresponds to Table 1. The revised statement now reads: "Moreover, Table 1 lists the calculated values of ten subjects' average arch stiffness in these three periods, along with their sex, mass, truncated foot length, normalized static arch height and maximum arch displacement."

3. In figure 4 description text, "The slopes of the three fitting lines were 0.124 kN/mm (Root Mean Square Error (RMSE)=1.589%) for the beginning of the heel contact phase, 0.0074 kN/mm (RMSE=0.211%) for the the plantar contact phase, and 0.038 kN/mm (RMSE=0.973%) for the end of the push-off phase". While in the figure picture, it was marked with the 0.0074kN/mm(RMSE=0.217). Is this a typo?

Response 3: *Thanks for your careful review and comments. This is a typo. We apologize for our mistake. We have made changes to the corresponding Figure 4 description text: "The slopes of the three fitting lines were 0.124 kN/mm (Root Mean Square Error (RMSE)=1.589%) for the beginning of the heel contact phase, 0.0074 kN/mm (RMSE=21.7%) for the plantar contact phase, and 0.038 kN/mm (RMSE=0.973%) for the end of the push-off phase".*

Response to Reviewer # 2

This paper proposes a multi-segment foot model to explore the forces on the foot arch and the variation of the foot arch linear stiffness during walking gait. The study has certain value, but there are still some issues that need to be addressed.

Thank you for your thorough review of this manuscript and for the time and effort you have invested in reviewing. This manuscript has been fully revised according to your professional suggestions.

1. How is the foot arch height calculated in the three phases of the stance phase?

Response 1*: We appreciate your careful review and valuable comments. In fact, the method for calculating foot arch height remains consistent across the three phases of the stance phase. As stated in Section* ***Kinematic and Kinetic Data of Foot****, on page 5, in the last paragraph: "As noted in Fig. 2(b), the navicular height was defined as the height of the arch of each participant."*

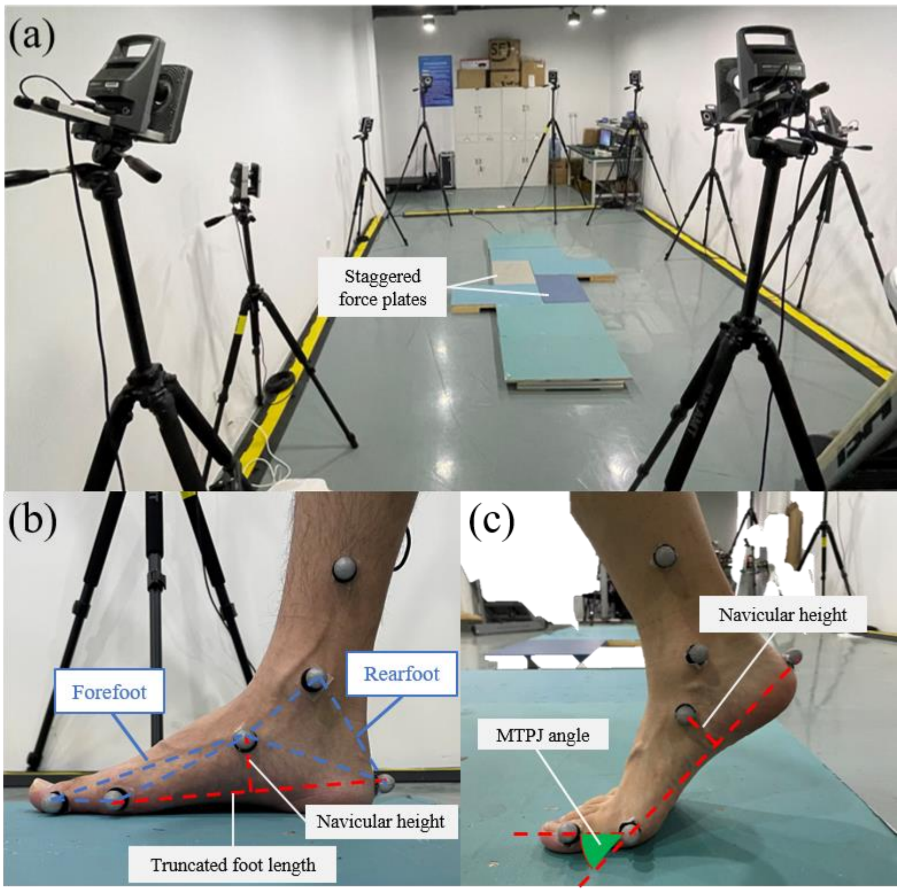

Fig.2 (a) The layout of the experimental site with a 10-infrared camera motion capture system and two force plates. (b) The location of the six markers at one foot and the definition of arch height. (c) The definition of MTPJ angle.

To enhance readability and facilitate understanding, we have added further clarification after that text: "After projecting the coordinates of all markers onto the proposed 2D plane, the foot arch height was calculated as the distance from the navicular bone marker to the line formed by the markers on the calcaneus and the first metatarsal head."

2. Validating the accuracy of the proposed model is crucial. This section needs to be added.

***Response 2**: Thank you for your critical comments. Like many other studies on foot arch stiffness [1-5], the model presented in this paper is designed to calculate, or estimate, the load on the foot arch. It is important to clarify that, similar to the reason why these references lack model accuracy validation, the absence of such validation in this study is not due to a lack of awareness of its importance. As mentioned in the paper, measuring the load on the foot arch in vivo during walking is extremely challenging. Additionally, using modeling software such as OpenSim for indirect calculations to validate the accuracy of our proposed model would be tangential to the main focus of this study.*

This explains why it is difficult to validate our model through direct experimental measurements or software simulations. Therefore, we can only validate the model's accuracy as best as possible by comparing the dynamic stiffness of the foot arch calculated by our model with results from existing studies. This serves as the accuracy validation section for our model. This approach is also the method used by D. A. Bruening et al. (2012, doi: 10.1016/j.gaitpost.2011.10.363) [6] in their study to validate model accuracy. The parameters selected for comparison include: the trend of changes in dynamic stiffness and the range of results. Moreover, we also calculate the coefficient of determination (R^2) between the force-displacement (or moment-angle) curves of the foot arch in previous studies and our work. The comparison of the trend in dynamic stiffness variation and the R^2 results represent the similarity in the variation of foot arch dynamic stiffness between this study and previous studies. The comparison of stiffness result ranges reflects the validity of the results in this study.

In the revised manuscript, we have made the following improvements:

*We added this clarification in **Abstract**: "By comparing the foot arch stiffness results with those from previous studies, the accuracy of the proposed model is indirectly validated."*

We also added this sentence in the last paragraph of Section ***Introduction****: "Comparing the foot arch stiffness results with those from previous studies provides indirect validation of the accuracy of the proposed model."*

Most importantly, we added a new Subsection ***Model Accuracy Validation Protocol*** *in the end of Section* ***Materials and methods****:*

"It is crucial to validate the accuracy of the proposed multi-segment foot model. The model established in this paper aims to calculate, or estimate, the load on the foot's arch. However, measuring the load on the foot arch during walking is highly challenging, and there is currently no standard protocol to validate the accuracy of foot biomechanics models. In this study, we validate the accuracy of our model by comparing the calculated dynamic stiffness of the foot arch with results from existing studies, including quasi-dynamic, dynamic, linear stiffness, and rotational stiffness studies. This comparison serves as the model validation method, similar to the approach used by Bruening et al. (2012) [23]. The parameters selected for comparison include: the trend of changes in dynamic stiffness and the range of results. Moreover, we also calculate the coefficient of determination (R^2) between the force-displacement (or moment-angle) curves of the foot arch in previous studies and our work. The comparison of the trend in dynamic stiffness variation and the R^2 results represent the similarity in the variation of foot arch dynamic stiffness between this study and previous studies. The comparison of stiffness result ranges reflects the validity of the results in this study.

Data from the force-displacement (or moment-angle) curves of the foot arch in previous studies were extracted using image data extraction software. These data were then compared with the results of this study. After aligning the data points through interpolation and scaling the values, the coefficient of determination (R^2) was calculated using standard statistical formulas to assess the similarity in the variation of foot arch stiffness. Quasi-dynamic and static foot measurement studies will be excluded from the comparison of the trend in dynamic stiffness variation and R^2 calculation. Studies with stiffness results in different units from this study will be excluded from the comparison of result ranges."

In the Section ***Results,*** *we also added a Table and a paragraph:*

"Table 2 presents the results of the model accuracy validation, comparing the trend and range of dynamic stiffness variation calculated by the model proposed in this study with those from previous studies, as well as calculating the coefficient of determination (R^2) between the force-displacement (or moment-angle) curves of the foot arch to characterize the similarity."

Table 2. **Comparison of the trend and range of dynamic stiffness variation calculated by the model proposed in this study with those from previous studies and the coefficient of determination (R^2) between the force-displacement (or moment-angle) curves**

Foot model	Trend of arch stiffness	Range of arch stiffness variation	Coefficient of determination (R^2)
This study	stiff-compliant-stiff-compliant	$0 - 0.188\ kN/mm$	–
Welte et al. (2021) [7]	–	$0.042 - 0.211\ kN/mm$	–
Sanchis-Sales et al. (2019) [14]	stiff-compliant-stiff-compliant	–	0.61
Kern et al. (2019) [15]	stiff-compliant-stiff-compliant	–	0.88
Venkadesan et al. (2020) [16]	–	$0 - 0.375\ kN/mm$	–
Davis et al. (2022) [17]	compliant-stiff-compliant	–	0.66
Kondo et al. (2021) [32]	stiff-compliant-stiff-compliant	–	0.74

– Not found or not applicable because it is a quasi-dynamic/static foot measurement or the units are different.

"As shown in Table 2, for studies with stiffness results in the same units as those defined in this study, the range of foot arch stiffness is generally between 0 and 0.2 kN/mm, which is consistent with the results of this study. For studies with different units but also calculating dynamic stiffness variations, the stiffness variation trends are largely consistent when viewed from the force/displacement curves. These studies all exhibit stiffness during the heel contact phase, compliance during the plantar contact phase, a sudden increase in stiffness, and compliance during the push-off phase. The R^2 values from curve comparisons indicate that these results all have R^2 values above 0.6, with the highest reaching 0.88. These comparative results support the validity of the model proposed in this study."

Finally, we stated the limitation of our lack of complete model accuracy validation in the end of Section ***Discussion****: "Finally, due to the lack of practical methods for measuring foot arch loads, the model accuracy validation in this study is incomplete. This limitation arises because the proposed model is specifically designed to calculate foot arch loads and cannot provide other mechanical quantities." and the potential future work regarding it: "Prospects also includes the potential to enhance the model by adding complexity, allowing for the calculation of other easily measurable physical quantities, thereby improving the accuracy validation method."*

3. What is the difference between calculating foot arch variation using foot arch angle changes versus using foot arch height changes?

Response 3*: We appreciate your professional comments. In a more general perspective, foot arch angle changes give insight into the angular geometry and alignment of the arch, while foot arch height changes directly measure the vertical displacement, providing a more tangible understanding of how the arch behaves under different conditions [7-9]. In clinical settings, measuring arch height can be more informative for diagnosing conditions like flat feet or assessing foot function in more*

detail [10, 11], while arch angle may be useful for evaluating overall foot posture and biomechanical alignment.

From the perspective of calculating foot arch variation, both methods are widely used, including studies using foot arch height changes [12, 13] and those using foot arch angle changes [2,3,4,5,14]. These references can also be found in the citations of this paper.

As shown in the literature, for easily measured conditions, such as cadaveric feet or stationary feet, vertical displacement is typically used to calculate stiffness, as this approach is more standardized. In contrast, for dynamic feet during gait, foot arch angle and forefoot-rearfoot angle are more commonly used to characterize foot arch stiffness.

This indirectly highlights the significance of the model proposed in this study, which aims to estimate the load on the foot arch during gait in vivo. One reason for using the moment-angle relationship of the foot arch to calculate stiffness is the simplicity of the method, as it only requires multiplying the plantar force of the forefoot/rearfoot by the lever arm (distance from the point of force application to the axis of rotation). However, this calculation represents the total moment acting on the foot rather than the load specifically borne by the foot arch.

4. In Table 2, the paper compares with other studies. In addition to differences in theoretical calculations, what are the differences in computational accuracy and results?

***Response 4**: Thank you for your careful review and critical comments. We have added the new **Table 2** that compares the trend and range of dynamic stiffness variation calculated by the model proposed in this study with those from previous studies, as well as calculating the coefficient of determination (R^2) between the force-displacement (or moment-angle) curves of the foot arch in the Section **Results**:*

"Table 2 presents the results of the model accuracy validation, comparing the trend and range of dynamic stiffness variation calculated by the model proposed in this study with those from previous studies, as well as calculating the coefficient of determination (R^2) between the force-displacement (or moment-angle) curves of the foot arch to characterize the similarity."

Table 2. Comparison of the trend and range of dynamic stiffness variation calculated by the model proposed in this study with those from previous studies and the coefficient of determination (R^2) between the force-displacement (or moment-angle) curves

Foot model	Trend of arch stiffness	Range of arch stiffness variation	Coefficient of determination (R^2)
This study	stiff-compliant-stiff-compliant	$0 - 0.188\ kN/mm$	–
Welte et al. (2021) [7]	–	$0.042 - 0.211\ kN/mm$	–
Sanchis-Sales et al. (2019) [14]	stiff-compliant-stiff-compliant	–	0.61
Kern et al. (2019) [15]	stiff-compliant-stiff-compliant	–	0.88
Venkadesan et al. (2020) [16]	–	$0 - 0.375\ kN/mm$	–
Davis et al. (2022) [17]	compliant-stiff-compliant	–	0.66
Kondo et al. (2021) [32]	stiff-compliant-stiff-compliant	–	0.74

– Not found or not applicable because it is a quasi-dynamic/static foot measurement or the units are different.

"As shown in Table 2, for studies with stiffness results in the same units as those defined in this study, the range of foot arch stiffness is generally between 0 and 0.2 kN/mm, which is consistent with the results of this study. For studies with different units but also calculating dynamic stiffness variations, the stiffness variation trends are largely consistent when viewed from the force/displacement curves. These studies all exhibit stiffness during the heel contact phase, compliance during the plantar contact phase, a sudden increase in stiffness, and compliance during the push-off phase. The R^2 values from curve comparisons indicate that these results all have R^2 values above 0.6, with the highest reaching 0.88. These comparative results support the validity of the model proposed in this study.".

The references in new Table 2 covers all those in the old Table 2. We also discussed the results in different references in the third paragraph of Section ***Discussion****: "Although the changes in linear arch stiffness during the stance phase have rarely been reported, we can still compare the arch stiffness results with those in the existing studies, as shown in Table 2."*

Reference:

[1] Bruening DA, Takahashi KZ. Partitioning ground reaction forces for multi-segment foot joint kinetics. Gait & Posture. 2018;62:111–116.

[2] Bruening DA, Cooney KM, Buczek FL. Analysis of a kinetic multi-segment foot model part II: Kinetics and clinical implications. Gait & Posture. 2012;35:535–540.

[3] Farris DJ, Kelly LA, Cresswell AG, Lichtwark GA. The functional importance of human foot muscles for bipedal locomotion. Proceedings of the National Academy of Sciences. 2019;116:1645–1650.

[4] Kern AM, Papachatzis N, Patterson JM, Bruening DA, Takahashi KZ. Ankle and midtarsal joint quasi-stiffness during walking with added mass. PeerJ. 2019;7:e7487.

[5] Davis DJ, Challis JH. Foot arch rigidity in walking: In vivo evidence for the contribution of metatarsophalangeal joint dorsiflexion. PLoS ONE. 2022;17:e0274141.

[6] Bruening DA, Cooney KM, Buczek FL. Analysis of a kinetic multi-segment foot model. Part I: Model repeatability and kinematic validity. Gait & Posture. 2012;35:529–534.

[7] Xiong S, Goonetilleke RS, Witana CP, Weerasinghe TW, Au EY. Foot arch characterization: a review, a new metric, and a comparison. J Am Podiatr Med Assoc. 2010 Jan-Feb;100(1):14-24. doi: 10.7547/1000014. PMID: 20093541.

[8] Dorsey S Williams, Irene S McClay, Measurements Used to Characterize the Foot and the Medial Longitudinal Arch: Reliability and Validity, Physical Therapy, Volume 80, Issue 9, 1 September 2000, Pages 864–871, https://doi.org/10.1093/ptj/80.9.864

[9] Wong CK, Weil R, de Boer E. Standardizing foot-type classification using arch index values. Physiother Can. 2012 Summer;64(3):280-3. doi: 10.3138/ptc.2011-40. PMID: 23729964; PMCID: PMC3396578.

[10] Charles, J. A., Mignot, C., & Jelinek, H. F. (2019). Reliability of a New Method to Determine Foot Arch Height for Clinicians. Journal of the American Podiatric Medical Association, 109(3), 187-192. Retrieved Jan 21, 2025, from https://doi.org/10.7547/18-007

[11] Juan C Zuil-Escobar, Carmen B Martínez-Cepa, Jose A Martín-Urrialde, Antonia Gómez-Conesa, Evaluating the Medial Longitudinal Arch of the Foot: Correlations, Reliability, and Accuracy in People With a Low Arch, Physical Therapy, Volume 99, Issue 3, March 2019, Pages 364–372, https://doi.org/10.1093/ptj/pzy149

[12] Welte L, Kelly LA, Lichtwark GA, Rainbow MJ. Influence of the windlass mechanism on arch-spring mechanics during dynamic foot arch deformation. Journal of The Roryal Society Interface. 2018;15:20180270.

[13] Venkadesan M, Yawar A, Eng CM, Dias MA, Singh DK, Tommasini SM. Stiffness of the human foot and evolution of the transverse arch. Nature. 2020;579:97–100.

[14] Sanchis-Sales E, Sancho-Bru JL, Roda-Sales A, Pascual-Huerta J. Variability of the Dynamic Stiffness of Foot Joints: Effect of Gait Speed. Journal of the American Podiatric Medical Association. 2019;109:291－298.
